# A cross-sectional study to identify the distribution and characteristics of licensed and unlicensed private drug shops in rural Eastern Uganda to inform an iCCM intervention to improve health outcomes for children under five years

**DOI:** 10.1371/journal.pone.0209641

**Published:** 2019-01-09

**Authors:** Denise Lynn Buchner, Freddy Eric Kitutu, Dónall Eoin Cross, Esther Nakamoga, Phyllis Awor

**Affiliations:** 1 Department of Community Health Sciences, Cumming School of Medicine, University of Calgary, Calgary, Alberta, Canada; 2 Pharmacy Department, Makerere University, Kampala, Uganda; 3 Department of Woman’s and Children’s Health, Upsala University, Uppsala, Sweden; 4 Institute of Biological, Environmental and Rural Sciences, Aberystwyth University, Wales, United Kingdom; 5 School of Public Health, Makerere University, Kampala, Uganda; Instituto Rene Rachou, BRAZIL

## Abstract

**Introduction:**

Malaria, pneumonia and diarrhea are leading causes of death in young children in Uganda. Between 50–60% of sick children receive treatment from the private sector, especially drug shops. There is an urgent need to improve quality of care and regulation of private drug shops in Uganda. This study was conducted to determine the distribution, the licensing status and characteristics of drug shops in four sub-districts of Kamuli district.

**Methods:**

This study was part of a pre-post cross sectional study that examined the implementation of an integrated Community Case Management (iCCM) intervention for common childhood illness in rural private drug shops in Kamuli District in Eastern Uganda. This mapping exercise used a snowball sampling technique to identify licensed and unlicensed drug shops and collect information about their characteristics. Data were collected using a questionnaire. GPS data were collected for all drug shops.

**Analysis:**

Quantitative data were analyzed using SPSS for descriptive statistics. Open ended questions were entered into NVivo 10 and analyzed using thematic analysis strategies.

**Results:**

In total, 215 drug shops in 284 villages were located. Of these, 123 (57%) were open and consented to an interview. Only 12 (10%) drug shops were licensed, 93 (76%) were unlicensed, and the licensing status of 18 (15%) was unknown. Most respondents were the owner of the drug shop (88%); most drug sellers reported their qualification as nursing assistants (70%). Drug sellers reported licensing fees and costs of contracting an “in-charge” as barriers to licensing. Nearly all drug shops sold drugs for malaria (91%) and antibiotics (79%).

## Introduction

Worldwide, the number of children who die below five years of age has decreased, although this decrease has been slow. In some countries, particularly in sub-Saharan Africa, the decrease has stagnated or even reversed [1]. In developing countries, pneumonia, malaria, and diarrhea remain main causes of mortality for children aged one to fifty-nine months [2, 3].

Even though effective and inexpensive treatments are available, many children in Africa, and especially those who live in rural or hard-to-reach places, do not receive life-saving treatments for common and easily treatable childhood illnesses [4–10]. In sub-Saharan Africa, only 39% of children with diarrhea receive oral rehydration salts (ORS) and 30% of children with presumed pneumonia receive antibiotics. Moreover, less than 20% of sub-Saharan children with fever receive artemisinin-based combination therapy [1].

In Uganda, private sector drug shops are often the first source of care for sick children [11–13], more so in rural and hard-to-reach communities [14–19]. The World Health Organization estimates that between 30–70% of febrile children are treated in the private sector, including drug shops [14]. Other studies report that between 39% and 53% of people who seek treatment for an illness consult a private health provider first, including general merchandise sellers, pharmacies, for-profit providers, and drug sellers [20–22]. In areas where access to formal health facilities is limited, private drug shops are often the first point of entry into the health system, particularly because care is accessible, affordable and perceived as friendly [23]. Moreover, poor quality of care at public health facilities, stock-out of essential drugs, long wait times, and a shortage of skilled providers also causes patients to consult private community-level providers, including drug shops [9, 24].

Despite widespread utilization of private sector drug shops, knowledge about the sector is limited. The evidence available suggests that many private sector providers are unlicensed, they diagnose illnesses incorrectly, and sell truncated doses of medicines, expired drugs, and drugs that are not recommended by national guidelines [17, 23].

## Ugandan context

### U5 mortality and morbidity

In Uganda, under-five mortality remains high at 53/1000, while the infant mortality rate is 28/1000. Under-five mortality in Uganda is caused primarily by pneumonia, diarrhea and malaria [25]. In rural areas, treatments for common childhood diseases are often inaccessible due to long distances to health facilities, and few or no trained health professionals available at the community level. These challenges are compounded by delayed health care seeking, poor health knowledge, irrational use of drugs, symptom overlap of conditions, co-morbid factors, low rates of immunization, malnutrition, under-dosing, low quality of drugs, and poorly trained health providers [26].

### Healthcare in Uganda

Utilization of public health facilities in Uganda is low. Konde-Lule et al [21] found that 42% of persons interviewed (n = 2580) reported being ill in the month prior to the survey but only 54% sought care from any type of provider. Of these, 37% visited a public health facility and 39% sought care from a private for-profit provider, often a clinic or drug shop. Similar results were found by Awor et al [27] who report that 53% of febrile children were taken to a private for profit health provider. The most commonly cited reasons for seeking health services from community-based private providers are convenience, and if the illness is perceived to be less severe [5, 28–31]. Many studies have called for strategies to regulate private drug providers [20, 21, 23, 27, 29, 32, 33].

### Private drug shops in Uganda

Despite high utilization rates, health services offered by private licensed drug shops in Uganda are poorly supervised and no supervision exists for unlicensed drug shops. In private licensed drug shops, standards for drug seller training are rarely adhered to, there are no opportunities for drug sellers to upgrade their skills and there are no data management requirements [34]. One study conducted exit interviews at licensed private drug shops in rural Uganda and found that only 10% of febrile children were given appropriate antimalarial treatment and 15% of children with cough and fast breathing received antibiotics. Moreover, only 14% of children who presented with diarrhea received ORS, and none received both ORS and zinc [27].

In Uganda, the National Drug Authority (NDA) controls the distribution of drug outlets through a system of registration that controls which health professionals are permitted to operate drug shops and where drug shops are located. A drug shop in Uganda can only be supervised by a pharmacy technician, registered or enrolled nurse, comprehensive nurse, registered or enrolled midwife, clinical officer of medical, psychiatric, orthopedic or dental discipline, or an anesthetic assistant [35].

Licensed drug shops in Uganda are licensed to sell a very restricted list of medicines, usually over-the-counter medicines, topical preparations and health supplies that can be applied without medical specialist supervision. However, Ministerial policy statements have extended legal permits for drug shops to include medicines used in treatment and control of disease that have major public health implication, such as Artemisinin-based combination therapies for management of malaria [35].

Drug shops in Uganda are required to meet minimum requirements for infrastructure including standards for walls, roof, floors, lighting, ventilation, toilet, shelves, cupboards and drawers, hand washing facility, external environment, and a minimum floor area of 16 square meters. Licensing fees in Kamuli municipality are 110,000 Ugandan shillings (38 USD) while fees for rural Kamuli district drug shops are 75,000 Ugandan shillings(26USD). In addition to the NDA licensing fee, a trading license fee of 90,000 Ugandan shillings (31USD) to 150,000 Ugandan shillings (51USD) is levied on drug shops by the local council where the drug shops operates [36, 37].

Evidence indicates that medicines for common illnesses such as malaria and other febrile illnesses are routinely provided in retail or general provision stores with fixed structures [38]. These medicines are also widely available from market stalls, mobile hawkers and other informal distribution points, which are often temporary structures. Many medicine outlets are unlicensed and may not be differentiated from retail or general provision shops.

## Study rationale

An intervention was planned for four sub-counties in Kamuli District. This intervention intended to 1) assist private drug shops to become licensed and 2) train drug sellers to distribute prepackaged, color-coded life-saving medicines to children under five years with fever, cough with fast breathing, and diarrhea using the integrated Community Case Management (iCCM) strategy. iCCM is a community-based intervention intended to be implemented through volunteer community health workers to improve adequate access to lifesaving medication [39]. While iCCM programs in Uganda have demonstrated success at increasing access to lifesaving medicines and saving lives [8, 40, 41], the majority of iCCM projects are dependent on donor funding; most iCCM projects end when the donor funding is finished [16, 39, 42]. This project intended to test a model of iCCM distribution through private drug sellers, which may be a more sustainable model.

To understand the landscape of drug shops in intervention areas, a mapping exercise was carried out in November 2014. The mapping exercise sought to understand the distribution, characteristics and location of private drug shops in intervention areas. This paper reports on results from the mapping exercise.

## Methods

### Setting

In November 2014, a cross-sectional mapping study was conducted to locate and collect data from all identified licensed and unlicensed drug shops in four sub-counties of Kamuli District (population 588,000) located in Eastern Uganda; Balawoli (pop 54,092), Kitayunjwa (pop 55,238), Namasgali (pop 37,524), and Butansi (pop 29, 799) [43]. This mapping exercise was part of baseline data collection for a larger pre-post cross-sectional study that identified and assisted unlicensed drug sellers to license their drug shops and trained up to two drug sellers per shop to provide iCCM to children under five years of age who sought treatment for malaria, cough with fast breathing, and diarrhea. A protocol that describes the larger study was previously published [44].

Kamuli District is primarily rural with few roads and limited infrastructure for health. There are two hospitals in the district, both located in main centers, three government health center III’s, 23 public health centers II’s, 25 private not-for-profit health center II’s and three private for profit health center II’s [45]. Most families in Kamuli District rely on subsistence agriculture and most have no access to electricity or running water.

### Sampling procedure

**Fig 1:**
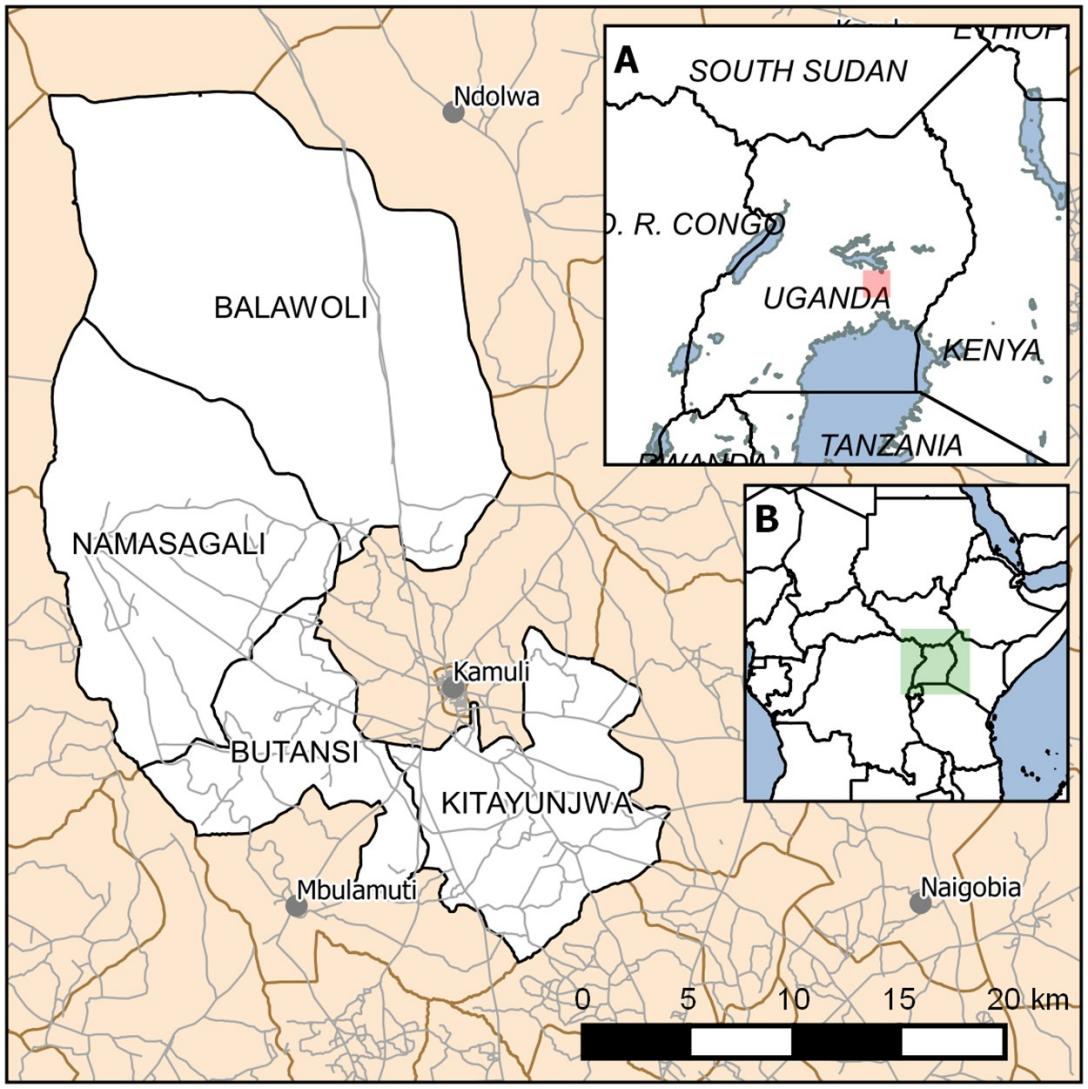
Sub-counties selected for drug shop mapping and surveys. Inset A indicates position of main map within Uganda. Inset B the position of Inset A within Africa. Data courtesy of GADM (gadm.org) and OpenStreetMap (openstreetmap.org: OpenStreetMap contributors).

An administrative list of district subdivisions was obtained from the Kamuli District Statistician. The list included 13 sub-counties, 79 parishes, and 756 villages. From this list, four sub-counties (Balawoli, Kitayunjwa, Namasgali, Butansi) were purposively selected based on consultations with district leadership. These sub-counties were selected due to high levels of poverty and poor access to health facilities; there are no hospitals or health centre IV’s in these sub-districts. Fig 1 illustrates sub-counties selected for the mapping exercise (Fig 1).

A snowball sampling technique was used to locate drug shops in selected sub-counties. A drug shop was defined as a shop with a physical location and structure whose main business was the sale of medications for human consumption. Starting at the edge of the first sub-county, a research assistant asked villagers for directions to the nearest village. Once in the village, the researcher explained the project and asked for directions to the nearest drug shop. The researcher had to win the confidence of drug sellers. Respondents were assured that their information was confidential and would not be reported to the National Drug Authority. The researcher moved around the village collecting data from every drug seller that consented to an interview and recording GPS data from every drug shop that was located, regardless of whether an interview was completed. When the researcher felt certain that she had located all the drug shops in the village, she asked for directions to the next village until all the villages identified on the sub-county list were located.

### Data collection

Data for this mapping exercise were collected over 30 days in November 2014. GPS data (latitude and longitude) were collected for all located drug shops and public health facilities in the selected sub-counties using a Tom Tom Go 630.

Drug sellers who were present on the day of mapping were approached and the study was explained. A local guide accompanied the research team during data collection; this individual assisted the research team to assure drug sellers that participation in the study would not jeopardize operation of the drug shop. As many unlicensed drug sellers fear an inspector who may confiscate drugs and close the shop, gaining trust from drug sellers was essential.

Drug sellers who consented were asked questions from a short survey that inquired about licensing status, characteristics of the drug shop, diagnostic tools available, and knowledge about iCCM (see supplementary material). The drug seller who was present on the day of mapping was asked to answer the survey, even if the respondent was not the owner of the drug shop. Drug sellers who reported that the drug shop was licensed were asked to show a valid licensing document. If the drug seller reported that the drug shop was licensed but could not produce a valid licensing certificate, the drug shop was coded as “unknown.” Drug sellers self-reported their qualifications, as most did not have certificates with them. Drug sellers were asked to report on iCCM medicines available for sale including 1) drugs for fever 2) drugs for fast breathing/cough and 3) drugs for diarrhea. The research team did not require drug sellers to show medicines for sale as doing so might dissuade drug sellers from responding candidly as many of them were already apprehensive. Finally, drug sellers were asked three open ended questions regarding challenges to becoming licensed. A comprehensive study of medicines, equipment, infrastructure and human resources available in selected drug shops was subsequently carried out as part of the intervention and will be reported in a separate manuscript.

### Data analysis

In order to examine the distribution of drug shops, GPS data were imported into QGIS (http://qgis.osgeo.org)(V2.8.1[46]) and maps of drug shop distribution were produced using freely available supplementary spatial data from GADM (gadm.org) and OpenStreetMap (openstreetmap.org).

Survey data were entered into REDCap (www.redcap.com) and then exported to IBM SPSS Statistics (v.22) and analyzed for frequencies and cross tabulations. Data from licensed and unlicensed drug shops (n = 12 and 93 respectively) were compared using Students T-tests.

Comments from open-ended questions were entered into Nvivo 10 and coded by themes that emerged from each question. Initial themes were reduced by finding similarities between themes.

## Results

### Distribution

Altogether, 215 drug shops in 284 villages in four sub-counties of Kamuli District were located. Drug shops were found to be significantly clustered with an observed mean distance of 335 meters between them, compared to an expected mean distance of 1123 meters if they were randomly distributed (nearest neighbor ratio 0.298; z = 19.550; p = <0.001).

### Licensing status

**Fig 2:**
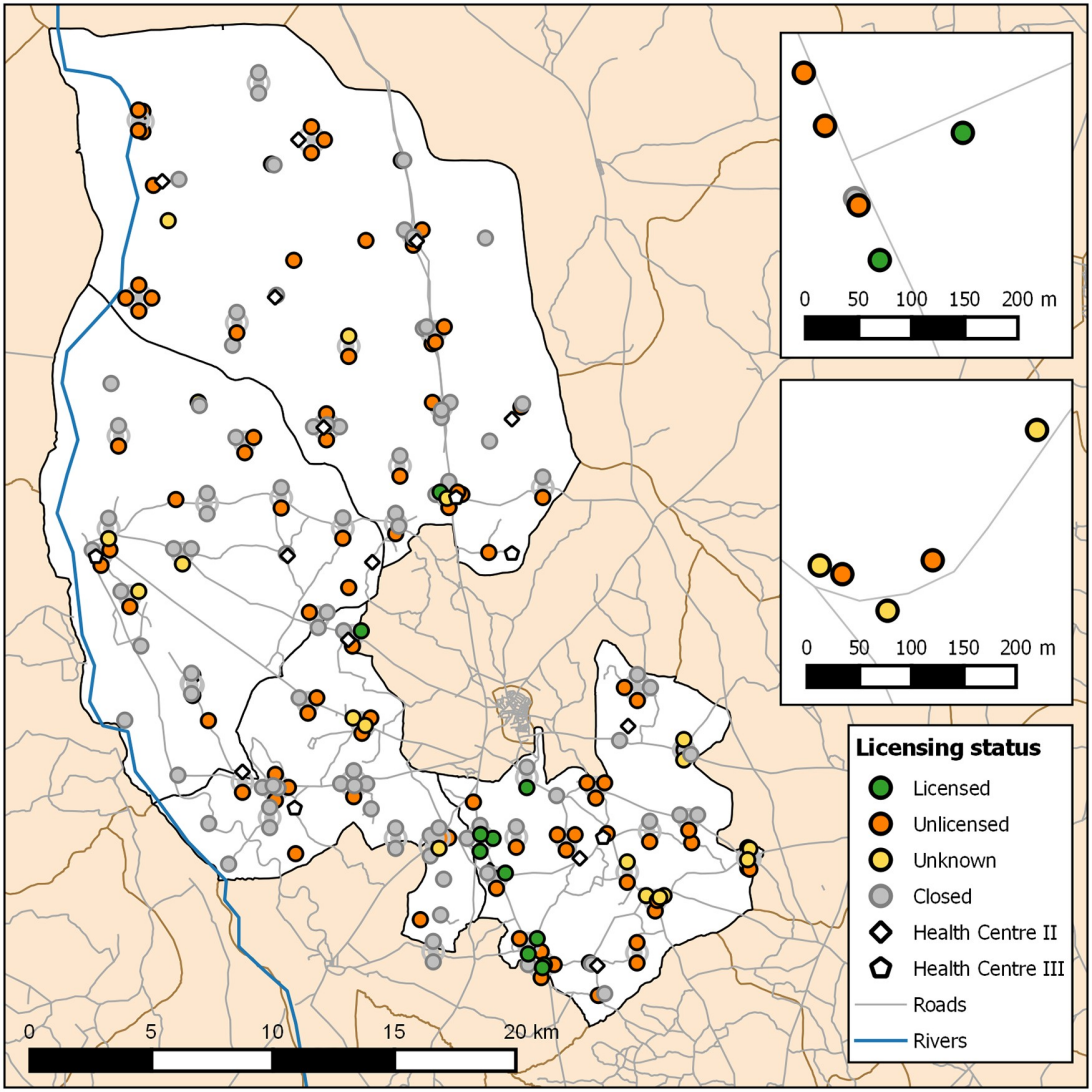
Licensing status of open drug shops and availability of health facilities in four sub-counties. Symbols of drug shops in very close proximity are displaced in an orbit around the actual location. Insets: representative close-ups illustrating the high degree of spatial clustering of drug shops. Data courtesy of OpenStreetMap (openstreetmap.org; OpenStreetMap contributors).

On the days of mapping, 123 (58%) drug shops were open and consented to an interview. Two (1%) drug shops were open but did not consent to an interview. Of the open drug shops that consented to an interview, 12 (10%) were licensed and 93 (76%) were not licensed. Eighteen (15%) reported a license but could not produce a valid license document (i.e. certificate, receipt); these drug shops are identified as “unknown” licensing status. Sixty-eight percent of open drug shops (n = 84) reported that the drug shop was never licensed. Fig 2 illustrates the distribution of open drug shops located in the four sub-counties (Fig 2).

### Length of operation

Regardless of licensing status, most (n = 80; 65%) drug shops had operated for more than one year. Only 11% (n = 14) of open drug shops had operated for less than 6 months and the majority of these (n = 11) were unlicensed. Only 42% (n = 5) of licensed drugs shops had operated for more than 3 years compared to 37% (n = 34) of unlicensed drug shops. There was no significant difference between length of operation of licensed and unlicensed drug shops (mean 71 and 48 months respectively; Student’s T = 1.036, df = 102, p = .302). Table 1 indicates length of operation for all open consenting drug shops.

**Table 1:** Length of drug shop operation.

Length	All (n = 123)	%	Licensed (n = 12)	%	Unlicensed (n = 93)	%	Unknown (18)	%
< 6 months	14	11	1	8	11	12	2	11
6 months–1 year	28	23	3	25	22	24	3	17
1–3 years	33	27	3	25	25	27	5	28
> 3 years	47	38	5	42	34	37	8	44
Unknown	1	1	0	-	1	1	0	-

### Drug shop workers

Most drug sellers (n = 89, 72%) working in the drug shop on the day of the survey reported that they were the owner or co-owner of the drug shop. Twenty-two drug sellers (18%) were employees, 11 (9%) a relative and one (1%) a friend. Table 2 indicates the status of the drug seller working in the drug shop on the day of the survey.

**Table 2:** Status of drug seller working in the drug shop.

Status	All (n = 123)	%	Licensed (n = 12)	%	Unlicensed (n = 93)	%	Unknown (n = 18)	%
Owner/ co-owner	89	72	8	67	69	74	12	67
Employee	22	18	3	25	15	16	4	22
Relative	11	9	1	8	8	9	2	11
Friend	1	1	0	-	1	1	0	-

Most drug sellers (69% n = 85) reported that the drug shop had one worker (Table 3). Thirty-three (27%) reported two workers, 3 (2%) three workers, 1 (1%) four workers, and 1 (1%) five workers. There was no significant difference between the number of workers employed in licensed and unlicensed drug shops (mean 1.7 and 1.3 respectively; Student’s t = 1.661, df = 103, p = 0.100).

**Table 3:** How many workers work in this drug shop?.

#	All (n = 123)	%	Licensed (n = 12)	%	Unlicensed (n = 93)	%	Unknown (n = 18)	%
One	85	69	5	42	70	75	10	5
Two	33	27	6	50	19	20	8	44
Three	3	2	1	8	2	2	0	-
Four	1	1	0	-	1	1	0	-
Five	1	1	0	-	1	1	0	-
Mean	1.37		1.66^a^		1.32^a^		1.4	

Values sharing a letter are not significantly different (p>0.05); comparison undertaken only between licensed and unlicensed drug shops (Student’s t-test).

### Drug seller characteristics

The study asked drug sellers to self-report their qualifications as most drug sellers did not have certificates with them. Most drug sellers (n = 86; 70%), regardless of licensing status, reported that they were “nursing assistants,” which in Uganda refers to a category of “health worker” who has acquired some knowledge and basic skill through an informal apprenticeship program. Table 4 outlines self-reported training of drug sellers working in open drug shops.

**Table 4:** Training of drug sellers present on day of mapping.

Training	All (n = 123)	%	Licensed (n = 12)	%	Unlicensed (n = 93)	%	Unknown (n = 18)	%
Nursing Assistant	86	70	8	67	65	70	13	72
Midwife	15	12	0	-	11	12	4	22
Nurse	12	10	3	25	9	10	0	-
Doctor	2	2	0	-	1	1	1	6
Other	8	7	1	8	7	8	0	-

We asked drug sellers about knowledge of signs of very sick children [10]. All drug sellers working in licensed drug shops (n = 12) could identify at least one sign of a very sick child compared to 89% (n = 83) of drug sellers working in unlicensed drug shops. Nine drug sellers (75%) working in licensed drug shops knew two signs of a very sick child and eight (67%) knew three signs. Sixty drug sellers (65%) working in unlicensed drug shops could identify two signs of a very sick child and 43 (46%) knew three. The most common known signs of a very sick child reported by drug sellers were convulsions (47%) and diarrhea (41%). Significantly more danger signs were identified by drug sellers in licensed than in unlicensed drug shops (3.8 vs 2.4, respectively; Student’s t = 2.539, df = 103, p = 0.013). Table 5 outlines drug seller self-reported knowledge about signs of a very sick child.

**Table 5:** Drug seller self-reported knowledge about signs of a very sick child.

Danger Sign	All (n = 123)	%	Licensed (n = 12)	%	Unlicensed (n = 93)	%	Unknown (n = 18)	%
Convulsions	58	47	8	67	40	43	10	56
Diarrhea	50	41	9	75	34	37	7	39
Dehydration	47	38	6	50	36	39	6	33
Vomiting	40	33	4	33	30	32	6	33
Difficult breathing	28	23	7	58	18	19	4	22
Very hot	25	20	3	25	19	20	3	17
Blood in stool	25	20	3	25	15	16	7	39
Unable to breastfeed	19	15	5	42	11	12	3	17
Very weak	16	13	3	25	12	13	1	6
Unconscious	15	12	3	25	10	11	2	11
Very small/ thin	7	6	0	-	6	6	1	6
Very cold	2	2	0	-	3	3	0	-
Red/bleeding umbilical cord	3	2	0	-	2	2	1	6
Mean total number of signs recognized	2.63		3.83^a^		2.43^b^		2.83	

Values not sharing a letter are significantly different (p<0.05); comparison undertaken only between licensed and unlicensed drug shops (Student’s t-test).

### Characteristics of drug shops

Most drug shops (n = 106, 86%) were attached to a house where people lived, often the drug seller and his/her family. Most drug shops (n = 111, 90%) sold only drugs. More licensed drug shops had reference material (n = 9, 75%) than unlicensed drug shops (n = 22, 24%). Five (42%) licensed drug shops had the Ugandan Clinical Guidelines while only five (5%) unlicensed drug shops had these guidelines. We analyzed reference material in drug shops by giving a score for each material held (iCCM guidelines, malaria, pneumonia, diarrhea; max score of 4) and found that licensed drug shops had significantly more reference material that unlicensed drug shops (mean score 1.58 vs. 0.39 respectively; Student’s t = 3.248, df = 12.318, p = 0.007).

Almost all drug shops had walls that were made from cement blocks (n = 121, 98%). One unlicensed drug shop had walls that were made from wood planks and another had walls made from bricks. Nearly all drug shops had floors made from cement (n = 119, 97%) and roofs made from iron sheets (n = 115, 93%).

Licensed drug shops also had significantly higher scores for infrastructure quality (mean score 7.33 vs. 6.38 respectively; Student’s t = 2.653, df = 103 and p = 0.009) based on one score given for each positive characteristic outlined in Table 6 (max score of 10).

**Table 6:** Characteristics of open drug shops on day of mapping.

Characteristic	All (n = 123)	%	Licensed (n = 12)	%	Unlicensed (n = 93)	%	Unknown (n = 18)	%
Door that locks	121	9	12	100	91	98	18	100
Drug storage free from moisture	118	96	11	92	90	97	17	94
Windows or vents that open	100	81	9	75	75	81	16	89
Rubber gloves	70	57	11	92	47	51	12	67
Electricity	43	35	9	75	26	28	8	44
Medicines stored on floor	11	9	0	-	11	12	0	-
Evidence of pests	7	6	0	-	6	7	1	6
Bed for patient to lie on	5	4	0	-	4	4	1	6
Direct sunlight on stored drugs	4	3	0	-	3	3	1	6
Refrigerator	1	1	0	-	1	1	0	-
Mean infrastructure score	6.58		7.33^a^		6.38^b^		7.11	

Values not sharing a letter are significantly different (p<0.05); comparison undertaken only between licensed and unlicensed drug shops (Student’s t-test).

### Drugs for sale

One hundred and fourteen drug sellers (11 licensed, 86 unlicensed, 17 unknown) provided information regarding drugs and diagnostic tests for sale. Most drug shops reported selling drugs for malaria (n = 104, 91%), antibiotics (n = 90, 79%), and ORS (n = 92, 81%). Table 7 outlines drugs for sale in open drug shops that provided information.

**Table 7:** Drugs for sale.

Drugs	All (n = 114)	%	Licensed (n = 11)	%	Unlicensed (n = 86)	%	Unknown (n = 17)	%
Drugs for malaria	104	91	11	100	76	88	17	100
Antibiotics	90	79	11	100	65	76	14	82
ORS	92	81	9	82	68	79	15	88
Zinc	64	56	8	73	42	49	14	82
RDT	13	11	3	27	4	5	6	35

### Open ended questions

Drug sellers were asked three open ended questions. These were: 1) What are some of the barriers this drug shop has faced with regards to licensing, 2) What would assist this drug shop to become licensed and 3) Are there any other challenges you face operating this drug shop? All drug sellers (n = 114) who consented to a survey answered at least one open ended question. Drug sellers provided short (2–3 sentences) responses to each question. The research assistant recorded the response verbatim on the survey form.

#### What are the barriers to becoming licensed?

Drug sellers overwhelming reported that the largest barrier to licensing the drug shop is the cost of the license, which was reported to cost between 100,000–120,000 Ugandan shillings (34-41USD). The second most commonly reported barrier was locating and paying for an “in-charge” to assist with the licensing process. As most drug sellers do not have the qualifications to apply for a license, those who wish to be licensed search for a qualified health professional who will apply on their behalf. This strategy for obtaining a license is not permitted in the NDA guidelines but is nonetheless a common practice [19]. Drug sellers interviewed for this study reported paying an in-charge between 150,000–500,000 Ugandan shillings (51-170USD) to assist with licensing the drug shop. Eight drug sellers reported the challenge of also having to pay a trading license fee. Interestingly, 12 drug sellers did not know that they required a license. Five drug sellers reported that they were unable to meet the requirements for licensing (i.e. handwashing, glass, drawers etc.) and three reported that the distance to the licensing office in Kamuli municipality was too far.

#### What would assist the drug shop to become licensed?

Fifty-four drug sellers reported that financial support or a reduction in the licensing fee would assist them to become licensed. Twenty drug sellers reported that a shorter licensing process or information about how to become licensed would help them license their shops. Eleven drug sellers asked for training so that they could qualify for a license. Five thought that the NDA should permit drug shops to become licensed without a medical supervisor. Other drug sellers asked for assistance with procurement of supplies and assistance to bring the drug shop up to required standards for infrastructure.

#### What other challenges have you faced?

Many drug sellers spoke about the challenges of operating a drug shop in a rural area where poverty is perceived to be pervasive. Moreover, most drug sellers reported being aware of selling wrong or partial doses of medicine but felt unable to stop the practice because customers demand certain medicines and could not always afford full doses.

Twenty-six drug sellers spoke about the challenge of procuring good quality and affordable drugs. These drug sellers reported purchasing drugs at retail prices in Kamuli municipality and then selling them to villagers with a price markup.

Thirteen drug sellers spoke about officials confiscating drugs. Seven drug sellers operating licensed drug shops complained about high competition due to many unlicensed drug shops operating nearby. Five drug sellers acknowledged that they sometimes treat people who are very sick even if they do not have the medicines or knowledge of how to treat them. Interestingly, one drug seller reported that she was struggling to operate her drug shop since a nearby health center closed as she no longer had anyone to get advice from.

## Discussion

These data indicate that drug shops are common in rural areas and most are unlicensed. Moreover, drug shops often operate in very close proximity to each other and sometimes near to public health facilities. Many studies report on the significant role of private drug shops in providing treatment for common childhood illnesses [12, 19, 27, 47, 48] but few have documented the distribution of drug shops in rural Uganda, or elsewhere. Villages where data for this study were collected were teaming with drug shops. Even the smallest village had numerous drug shops, often operating steps from each other. Maps drawn from GPS data collected for this study were challenging to create as coordinates were often only marginally apart.

Data from this study found that 76% of drugs shops were unlicensed; these data are similar to census data reported by Konde-Lule [21] where 77% of drug shops were unlicensed and lower than data reported by Weng [38] who identified 48 drug shops in one sub-county in Uganda, of which only 1 (2.1%) self-identified as licensed. Our study required drug sellers to provide a valid licensing certificate; if the certificate was expired, reported to be under renewal, or if the drug seller was unable to locate it, then the drug shop was classified as “unknown.” Possibly, the proportion of drug shops that were unlicensed was higher than we report due to the number of drug shops with “unknown” licensing status. Large numbers of unlicensed drug shops reported by this study and others is reason for concern as they operate without any oversight from formal authorities.

Wang et al. [38] report that drug shops have advantages over public health facilities, and especially in the rural context. These authors report that drug shops are closer to village centres, have shorter wait times, provide flexible operating hours and have fewer drug stock outs. Other studies report that private sector drug shops are perceived as accessible, affordable and friendly to nearby villagers [9, 23, 24]. Our study shows that private drug shops routinely operate near to public health facilities, indicating that the distance to a public health facility may not predict the establishment of private drug shops. While our study did not set out to understand what motivates people to seek care from private drug sellers, our data indicate that drug shops operating near to public health facilities are viable businesses. Indeed, one participant in our study reported feelings of loss when a nearby public health facility closed as she could no longer seek advice from health centre staff when a patient with challenging needs sought care from her unlicensed drug shop. It is evident that licensed and unlicensed drug shops exist in a health care system that is poorly understood. Unlicensed private drug shops far outnumber private licensed and public health facilities combined yet their place in the system is largely unrecognized or understood.

Our study provides a rare perspective about unlicensed drug shops in the sub-counties where data were collected. Drug sellers operating from unlicensed drugs shops overwhelmingly reported licensing fees and challenges engaging an in-charge as major barriers to licensing their drug shops. These data indicate that drug sellers may wish to operate legal licensed drug shops but lack capacity and resources to do so; they may be willing to work within the system if strategies to engage these drug sellers, improve skills and update infrastructure can be identified.

Many studies report widespread concern with the operation of unlicensed drug shops including selling expired or ineffective drugs [38] and inappropriate treatments [9, 23]. Data from this study provide additional evidence to support these concerns. In this study, staff in licensed drug shops recognized significantly more danger signs for sick children than did staff working in unlicensed drug shops. Data from other studies have found that level of training and education is positively correlated with ability of health providers to correctly identify and treat common illnesses [17, 18, 49].

In this study, more drug sellers working in licensed drug shops reported their cadre as “nurse” (25%) compared to unlicensed drug shops (10%), indicating that drug sellers working in licensed drug shops may have more knowledge and training than staff in unlicensed drug shops and are therefore more able to correctly identify danger signs in young children.

However, despite the positive correlation between licensing status and ability to identify danger signs in young children, drug sellers in all located drug shops had despairingly low levels of knowledge about danger signs in young children. Most drug sellers in this study (70%) reported their cadre as “nursing assistant.” In Uganda, “nursing assistant” is an ambiguous description that describes a wide spectrum of experience and/or training. It is not known if nursing assistants working in licensed drug shops had more training or mentorship than nursing assistants working in unlicensed drug shops and if this might explain higher levels of knowledge related to identification of danger signs for very sick children. More in-depth studies on skills of drug sellers would improve knowledge of quality of care in these shops and how it might be improved.

In this study, drug sellers self-reported drugs for sale as many were selling drugs illegally in unlicensed drug shops. Consequently, our data likely underreports medicines for sale as drug sellers could choose to not disclose drugs for sale in their shops. In this stud, 88% of drug sellers self-reported selling drugs for malaria and 75% self-reported selling antibiotics. These data are alarming and especially when contextualized by the low level of training of most drug sellers operating from unlicensed drug shops and the complete non-regulation of these shops. These data, which report on drugs for sale in unlicensed drug shops, are rare. In Tanzania, Hetzel [49] studied drug seller knowledge and distribution of malaria medications and noted that drug shops, including drug shops and general shops, routinely sold prescription medicines (antimalarials, antibiotics) that are not permitted according to the licensing standards.

## Conclusion

Drug shops in intervention districts were common and clustered near to each other. Most drug shops were unlicensed. Most drug sellers in licensed and unlicensed drug shops reported their cadre as, “nursing assistant” (70%). Drug sellers working in licensed drug shops recognized significantly more danger signs for sick children than did drug sellers working in unlicensed drug shops. Most drug shops, regardless of licensing status, reported selling drugs for malaria and antibiotics. Overwhelmingly, drug sellers reported barriers to licensing their shop due to cost and the inability to secure an “in-charge.” There is an urgent need to regulate the operation of private drug shops in rural Uganda.

## Supporting information

S1 File (CSV)
